# A novel cascade machine learning pipeline for Alzheimer’s disease identification and prediction

**DOI:** 10.3389/fnagi.2022.1073909

**Published:** 2023-01-16

**Authors:** Kun Zhou, Sirong Piao, Xiao Liu, Xiao Luo, Hongyi Chen, Rui Xiang, Daoying Geng

**Affiliations:** ^1^Academy for Engineering and Technology, Fudan University, Shanghai, China; ^2^Department of Radiology, Huashan Hospital, Fudan University, Shanghai, China; ^3^School of Computer and Information Technology, Beijing Jiaotong University, Beijing, China

**Keywords:** Alzheimer’s disease, coronal T1 weighted images, machine learning, automatic segmentation, radiomics classification

## Abstract

**Introduction:**

Alzheimer’s disease (AD) is a progressive and irreversible brain degenerative disorder early. Among all diagnostic strategies, hippocampal atrophy is considered a promising diagnostic method. In order to proactively detect patients with early Alzheimer’s disease, we built an Alzheimer’s segmentation and classification (AL-SCF) pipeline based on machine learning.

**Methods:**

In our study, we collected coronal T1 weighted images that include 187 patients with AD and 230 normal controls (NCs). Our pipeline began with the segmentation of the hippocampus by using a modified U2-net. Subsequently, we extracted 851 radiomics features and selected 37 features most relevant to AD by the Hierarchical clustering method and Least Absolute Shrinkage and Selection Operator (LASSO) algorithm. At last, four classifiers were implemented to distinguish AD from NCs, and the performance of the models was evaluated by accuracy, specificity, sensitivity, and area under the curve.

**Results:**

Our proposed pipeline showed excellent discriminative performance of classification with AD vs NC in the training set (AUC=0.97, 95% CI: (0.96-0.98)). The model was also verified in the validation set with Dice=0.93 for segmentation and accuracy=0.95 for classification.

**Discussion:**

The AL-SCF pipeline can automate the process from segmentation to classification, which may assist doctors with AD diagnosis and develop individualized medical plans for AD in clinical practice.

## Introduction

Alzheimer’s disease (AD) is a progressive, irreversible degenerative brain disease characterized by memory loss and cognitive impairment (Sevigny et al., 2016; Li et al., 2019). Detecting AD at an earlier or prodromal stage is vital for preventing disease progression. Neuroimaging biomarkers may give more predictive information and make the diagnosis more reliable (Márquez and Yassa, 2019). Magnetic resonance imaging (MRI) is a type of brain imaging biomarker that can be used to detect the structures in brain volume, such as hippocampus atrophy or ventricle enlargement, and therefore the onset of Alzheimer’s disease and distinguishing normal controls (NC) from AD (Jack Jr et al., 2011; Hosseini-Asl et al., 2016).

Nowadays, machine learning and radiomics features analysis have been employed to analyze MRI data for identifying accurate biomarkers of AD (Moradi et al., 2015; Pellegrini et al., 2018; Feng et al., 2021). The most widely used classification techniques are support vector machine (SVM), artificial neural network (ANN), and deep learning. The main difference between SVM and ANN is the optimization problem. Compared with ANN, SVM is more generalizable and can obtain global optimal solutions. Among them, feature extraction is an important step (Anami and Elemmi, 2022). It is useful to combine neural networks and intelligent agents for medical image analysis according to the research of Shi et al. (Sarvamangala and Kulkarni, 2022). However, deep learning can be trained to automatically extract features from target regions without intervention (Shen et al., 2017; Chen et al., 2022). When the number of available samples is large enough, deep learning will be highly effective (Shen et al., 2017).

In recent years, researchers provided analysis of the works done using machine learning for Alzheimer’s disease. For instance, Gray et al. (2013) proposed a multi-modality classification framework derived from random forest and evaluated the framework by application to neuroimaging and biological data from the Alzheimer’s Disease Neuroimaging Initiative. Feng et al. (2021) developed three classification models to discriminate AD, mild cognitive impairment (MCI), and NC patients by extracting and analyzing 3,360 radiological features from 3D T1-weighted magnetization-prepared rapid gradient echo images. Cao et al. (2017) proposed a multicore-based downsampling and oversampling method. The sparsity of regions of interest (ROI) was achieved using marginal Fisher analysis with ℓ2.1-multiple kernel learning based on paradigms, which enabled simultaneously select subsets of relevant brain regions and improved the accuracy of AD classification. Besides, In the study of Zhou et al. (2018), Kruthika et al. (2019), Peng et al. (2019), Lin et al. (2020), and Richhariya et al. (2020), the researchers used multistage classifier, SVM, recursive feature elimination, and TrAdaBoost methods to analyze and identify AD based on MRI images, and the classification accuracy is 0.88, 0.97, 0.89, and 0.94, respectively.

Deep learning models, including convolutional neural networks (CNN), have increasingly been used for image analysis and computer vision. In the field of medical imaging, deep learning-based medical imaging applications outperform traditional methods in complex tasks (Duraisamy et al., 2019; Singh et al., 2020). Of all brain regions associated with AD, the hippocampus is one of the earliest to undergo pathology changes (Han et al., 2014; Dhikav et al., 2017; Park et al., 2022). Helaly et al. (2022) proposed a deep learning Alzheimer’s disease hippocampus segmentation framework (DL-AHS) for hippocampal segmentation to detect and identify AD. In addition, the data was augmented using deep convolutional generative adversarial networks (DC-GAN). Sun et al. (2020) proposed a V-Net-based 3D CNN to segment the bilateral hippocampus from 3D brain MRI scans and diagnosed AD progression status. Kwak et al. (2022) constructed a deep convolutional neural network to classify stable and progressive MCI and evaluated the relative contribution of each hippocampal subfield. Buvaneswari and Gayathri (2021) used the seven morphological features like grey matter, white matter, cortex surface, gyri and sulci contour, cortex thickness, hippocampus and cerebrospinal fluid to accurately classify AD and dementia conditions.

As mentioned above, promising progress has been made in AD prediction studies based on structural radiological features and machine learning. However, most studies have focused on the 3D T1 sequence, which has high resolution with long acquisition times. However, coronal T1 weighted images are more commonly applied in clinical practice. In addition, current classification algorithms are based on semi-automatic or manual marking of ROI, and there is no automatic framework from segmentation to classification. Nevertheless, a fast and accurate identification framework is extremely important for the early diagnosis of AD in routine clinical work.

Motivated by these properties and important results, we devised a contribution to the study of AD using coronal T1 weighted images. We first migrated the U^2^-net to the hippocampus segmentation task and added a deep supervision mechanism module to improve the model performance. Second, we applied different feature extraction methods to extract features from the segmented hippocampal regions and analyzed which features were more relevant to AD classification. Finally, we performed a binary classification task to detect if patients are healthy or have dementia on our datasets and explored the possibility of applying our model to clinical practice.

## Materials and methods

**Figure 1:**
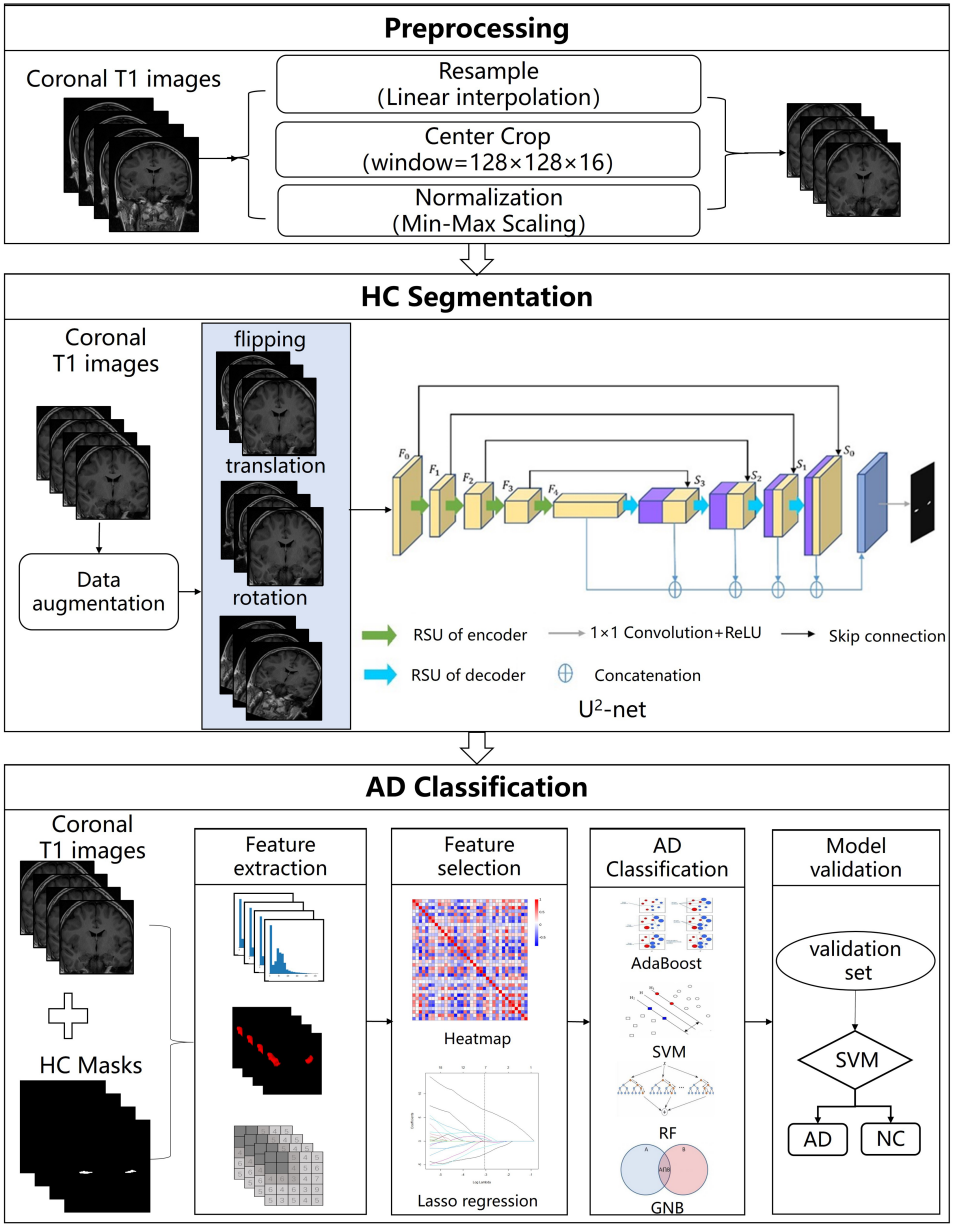
The structure of Alzheimer’s segmentation and classification (AL-SCF). This study is mainly divided into three parts. The first part is data collection and preprocessing, the second part is hippocampus segmentation, and the third part is Alzheimer’s disease (AD) classification.

The AL-SCF pipeline workflow includes the MRI preprocessing, the hippocampus segmentation, and the AD classification model. All the specific processes of the pipeline are shown in Figure 1.

### Patients

In total, 187AD patients aged 50–75 years were retrospectively selected from the electronic medical records at Huashan Hospital, Shanghai, China from April 2016 to 2021. The patient’s demographic information is provided in Table 1.

**Table 1:** Patient demographic information.

Variables	AD group	NC group	Value of *p*
Sample size	187	230	/
Age (years, mean ± SD)	67.45 ± 11.23	65.56 ± 13.42	0.38
Gender (Male: Female)	23:27	30:20	0.16
Education (years, mean ± SD)	15.00 ± 3.00	15.60 ± 3.00	0.52

AD, Alzheimer’s disease; NC, normal control; SD, standard deviation.

All the AD patients were diagnosed by a qualified neurologist using criteria for amnestic AD Fennema-Notestine et al. (2009), with mini-mental state examination (MMSE) scores between 12 and 27 (inclusive) and clinical dementia rating (CDR) scores of 1 or 2. The inclusion criteria of AD patients were as follows: (1) right-handed Han Chinese patients older than 50 years; (2) clinically diagnosed as AD confirmed by qualified neurologists; (3) with available hippocampus MRI images. The exclusion criteria of AD patients included: (1) presented structural abnormalities such as cortical infarction, tumor, or subdural hematoma; (2) low-quality MRI scanning. 230 age and sex-matched normal controls (NC) with hippocampus MRI images were also enrolled.

All participants were scanned using a standard eight-channel head coil on a 3 Tesla MR scanner (Discovery 750, GE Healthcare, United States). Foam pads and headphones were used to minimize participants’ head motion and scanner noise. In the model construction, coronal T1 weighted images were used. The parameters were as follows: repetition time (TR) = 225 ms, echo time (TE) = 24 ms, inversion time (TI) = 780 ms, slice thickness = 4 mm. The field of view was 240 mm × 240 mm, and the matrix was 512 × 512, resulting in the in-plane resolution of 0.47 mm × 0.47 mm.

### Hippocampus annotation

All MRI images were first resampled to voxel size 1 mm × 1 mm × 1 mm with a resolution of 224 × 224 × 16 by linear interpolation method. Then, we performed the image grayscale normalization with the image grayscale unified adjustment to [0,255] and cropped all images to 128 × 128 × 16.

Hippocampus was annotated manually by two neuroradiologists, both with 5 years of experience using ITK-SNAP software.^1^ All segmentation results were corrected by a senior neuroradiologist with 15 years of clinical experience. In this study, the dataset was randomly divided into the training set (*n* = 334) and test set (*n* = 83) with a ratio of 8:2.

### Segmentation model

The segmentation model consists of an encoder and a decoder, which is a two-level nested structure, and the top level is a large U-shaped structure consisting of five stages (Qin et al., 2020). Specifically, in the decoder of our model, a deep supervision mechanism (Lee et al., 2015) was used to improve the performance of our model. It can upsample the feature maps to the same size and fuse them to generate the final segmentation results with a concatenation operation followed by a 1 × 1 convolution layer.

For training the segmentation model, the epoch was set to 250 and the batch size was 12. After applying augmentation techniques containing flipping, translation, and rotation to the training set, the number of data samples increased to 668. We used the Adam optimizer to update the gradient with an initial learning rate of 0.001 and momentum β_1 = 0.9. The experiments were deployed on NVIDIA Titan GPU with the Pytorch^2^ framework.

### Classification model

#### Feature extraction

Radiomics features were extracted by Pyradiomics (Van Griethuysen et al., 2017). According to guidelines from the Image Biomarker Standardization Initiative (IBSI; Zwanenburg et al., 2020), the extracted radiomics features included 140 first-order features, 175 morphological features, and 501 higher-order texture features, involving Gray Level Co-occurrence Matrix (GLCM), Gray Level Size Zone Matrix (GLSZM), Gray Level Run Length Matrix (GLRLM), Neighbouring Gray Tone Difference Matrix (NGTDM), and Gray Level Dependence Matrix (GLDM).

#### Feature selection and classification models

The hierarchical clustering method (Murtagh and Contreras, 2012) and the Least Absolute Shrinkage and Selection Operator (LASSO) algorithm (Roth, 2004) were performed to reduce the dimensionality of features. First, hierarchical clustering was used by calculating Spearman’s correlation matrix of the extracted features and the highly correlated features should be removed. Subsequently, LASSO was applied to select representative features and remove irrelevant and redundant features which would degrade the performance of the further process. Moreover, Gaussian Naive Bayes (GNB; Kohavi, 1996), random forest (RF; Breiman, 2001), support vector machine (SVM; Graves, 2012), and AdaBoost (Dietterich, 2000) classifiers were implemented in the AD classification task.

### Evaluation metrics

Dice coefficient is a statistical metric that measures the similarity between ground truth and segmentation results, which is defined as follows:


(1) $$\mathrm{Dice}=\frac{2\sum_{i=1}^{N}p_{i}q_{i}}{\sum_{i=1}^{N}p_{i}^{2}+\sum_{i=1}^{N}q_{i}^{2}}\;$$

where *N* is the number of total pixels on the segmentation results; $p_{i}$ and $q_{i}$ are the pixels of predicted segmentation result and the ground truth, respectively. For example, Dice=1 means two samples are exactly overlapping.

The diagnostic performance of the classification model was assessed using receiver operation characteristics (ROC) curve analysis and measured by area under the ROC curve (AUC). Then, we defined AD patients as positive samples and obtained the calculation formulas of accuracy (ACC), sensitivity (SEN), and specificity (SPE) by the confusion matrix:


(2) $$\{\begin{array}{c} ACC=\frac{TP+TN}{TP+TN+FP+FN}\\ SEN=\frac{TP}{TP+FN}\\ SPE=\frac{TN}{TN+FP} \end{array}$$

### Statistical analysis

In statistical tests of demographical and clinical characteristics, the Mann–Whitney *U*-test was used for numerical variables, and Fisher’s exact test was used for categorical variables. Statistical analyses were performed using SPSS (version 22.0, IBM). In addition, feature selection and radiomics signature construction and validation of the classification model were conducted using scikit-learn packages^3^ based on Python (Version 3.8.0; https://www.python.org).

## Results

### Segmentation results

**Figure 2:**
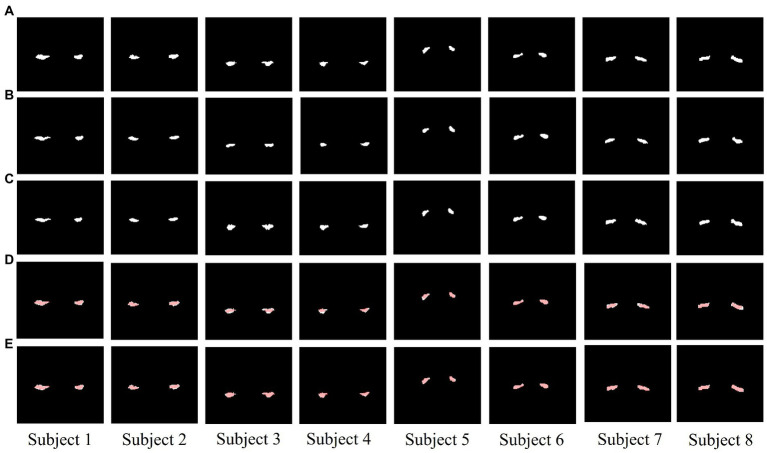
The segmentation results of U^2^-net **(A–E)**. **(A)** ground truth (GT). **(B)** Segmentation results before data augmentation. **(C)** Segmentation results after data augmentation. **(D)** The difference between GT and segmentation results before data augmentation (red areas are the segmentation results). **(E)** The difference between GT and segmentation results after data augmentation (red areas are the segmentation results). Columns from left to right are the samples (Subject 1–8).

A total of 668 MRI images along with their corresponding hippocampal binary masks were taken as the training set to fit into the segmentation model. Then, the model was tested for another 83 MRI images and the segmentation results are shown in Figure 2. Figure 2A are some of the sample ground-truth, and the corresponding segmentation results before and after data augmentation are shown in Figures 2B,C. It is observed that the segmentation results after data augmentation are much more identical to their corresponding ground-truth images from Figures 2D,E.

**Figure 3:**
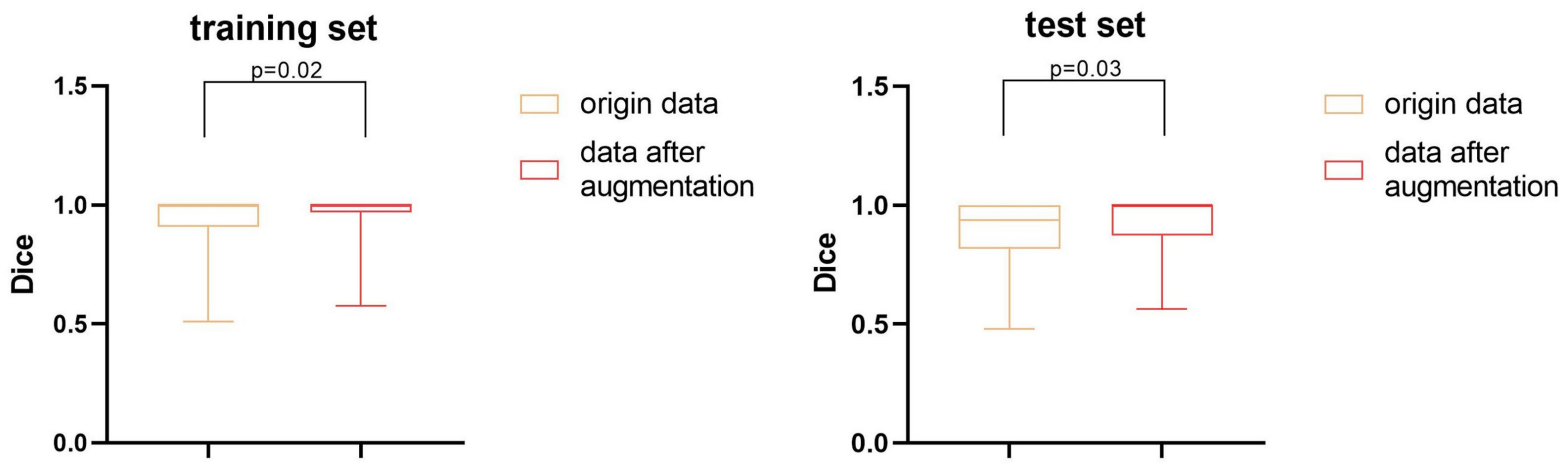
The distribution of Dice coefficients of segmentation results.

We also performed a quantitative analysis of the segmentation results. Table 2 shows that the predicted segmentation results achieved Dice = 0.95 ± 0.03 in the training set and Dice = 0.90 ± 0.02 in the test set before data augmentation. The final Dice = 0.93 ± 0.01 in our test set after data augmentation, which was significantly higher (*p* < 0.05) than when no data augmentation was applied at a cost of several seconds. Figure 3 shows the distribution of segmentation results in training and test sets. It is noted that the results are more concentrated and better for more difficult segmented images when applying data enhancement.

**Table 2:** Dice coefficients of segmentation models in the training and test sets.

Dataset	Origin data	Data after augmentation	Value of *p*
Training	0.946±0.03	**0.973** ± **0.02**	**0.02**
Test	0.895±0.02	**0.929** ± **0.01**	**0.03**

The best results are in bold.

### Classification results

**Figure 4:**
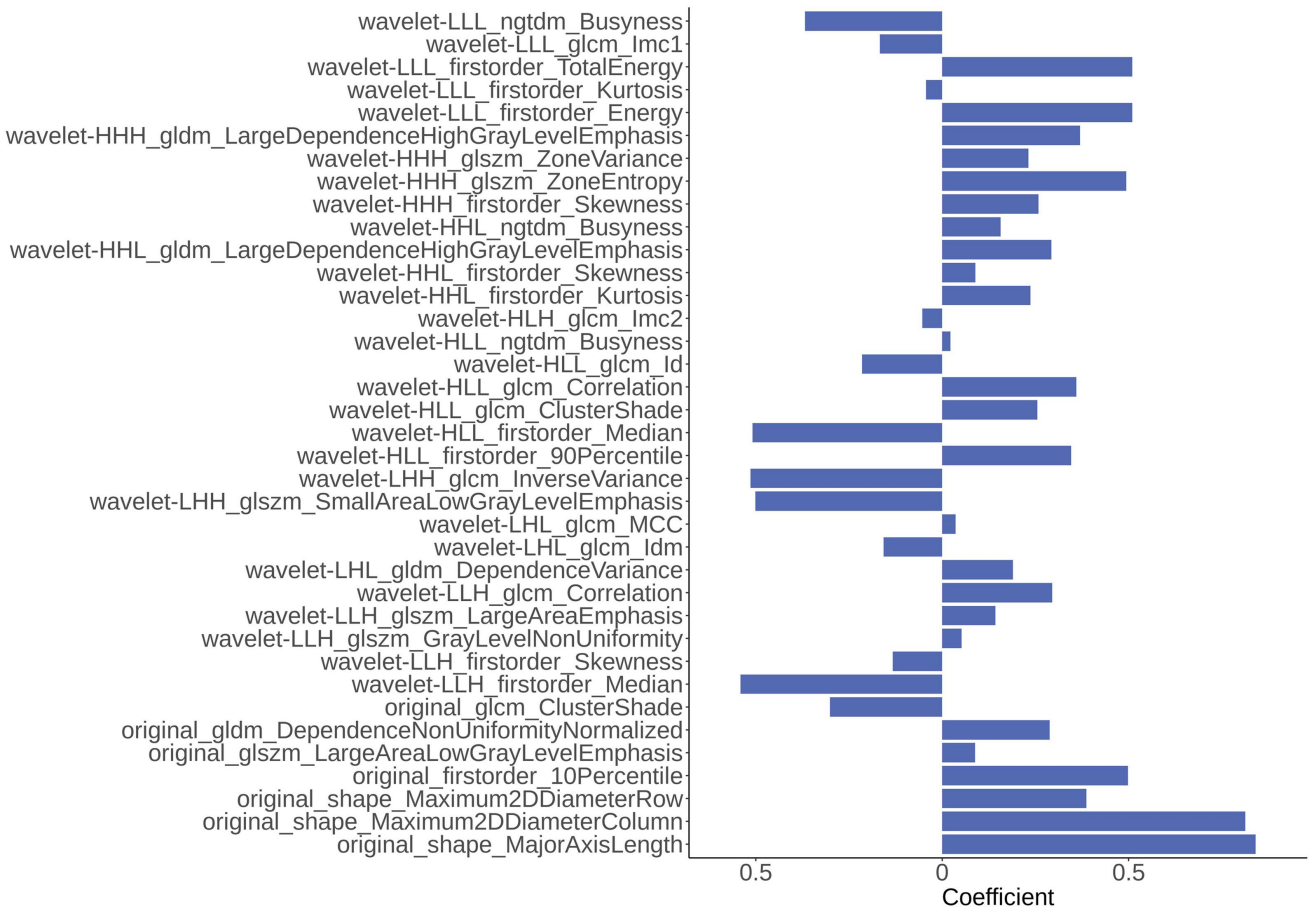
The importance of 37 radiomics features generated from Least Absolute Shrinkage and Selection Operator (LASSO).

In the part of feature extraction, 851 radiomics features were extracted for the region of the hippocampus. After using Spearman’s correlation matrix filter, 727 features remained. Then, we used the LASSO filter with 5-fold cross-validation to select 37 features for the AD classification task. The importance of 37 features were determined with the weights of LASSO, which was shown in Figure 4. It is illustrated that the shape_MajorAxisLength feature has the largest importance value of 0.84, followed by the shape_Maximum2DDiameterColumn feature with an importance value of 0.81.

**Figure 5:**
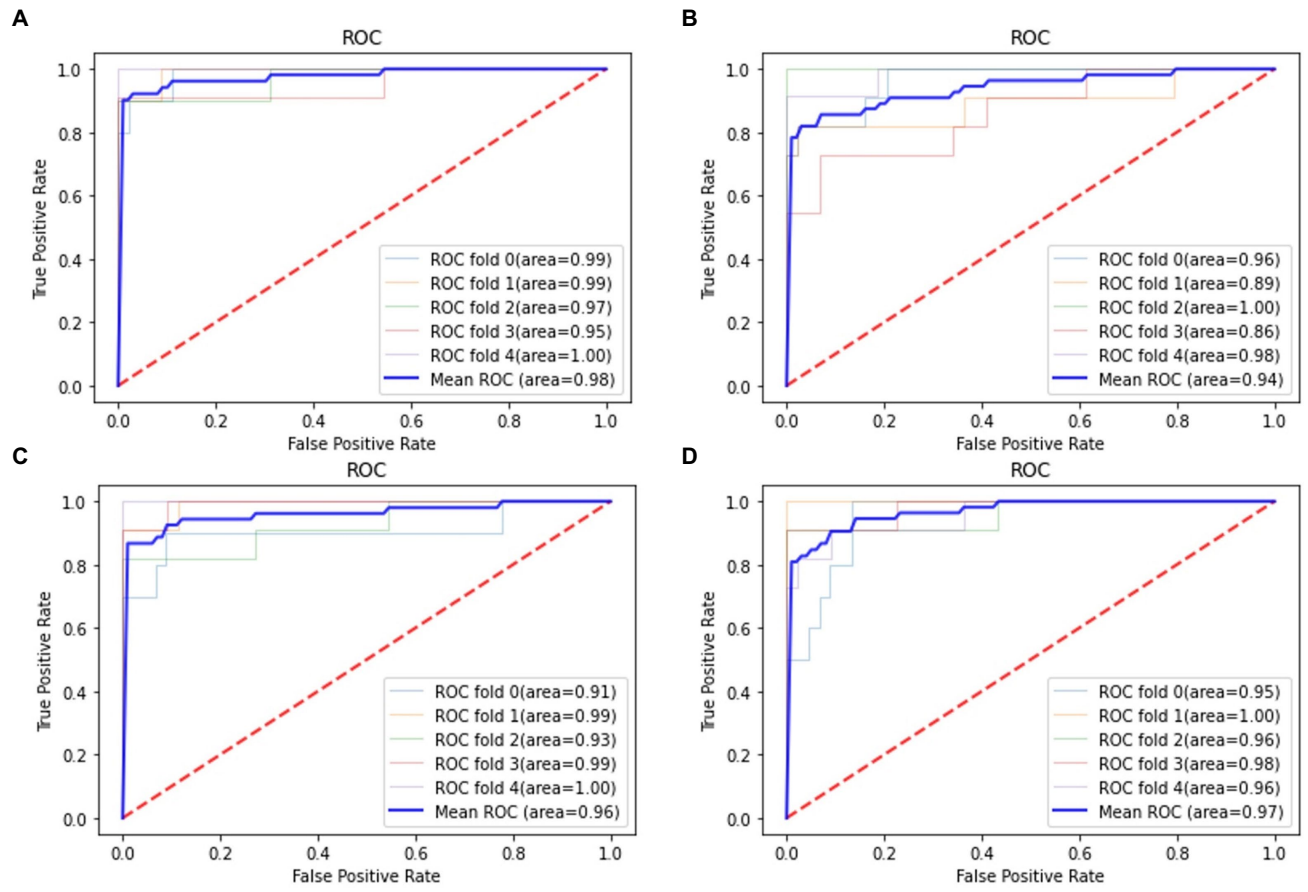
Receiver operation characteristics (ROC) curves for AD classification in the training set. **(A)** Support vector machine (SVM) classifier; **(B)** RF classifier; **(C)** GNB classifier; **(D)** Adaboost classifier.

For all classification models, we used 5-fold cross-validation to make the results more reliable. Table 3 and Figure 5 show the classification results and ROC curves for AD classification in the training set. Among the four classifiers, the SVM classifier achieved the best performance in the classification task, with ACC = 0.97 (95%CI: 0.95–0.98), SEN = 0.97 (95%CI: 0.94–0.98), SPE = 0.96 (95%CI: 0.95–0.99), and AUC = 0.98 (95%CI: 0.96–1.00).

**Table 3:** Classification results of different classifiers in the training set.

Classifier	ACC	SEN	SPE	AUC
SVM	**0.97**	**0.97**	0.96	**0.98**
	**(0.96, 0.98)**	**(0.95, 0.99)**	(0.93, 0.99)	**(0.96, 1.00)**
RF	0.86	0.89	0.93	0.94
	(0.83, 0.89)	(0.86, 0.92)	(0.91, 0.95)	(0.92, 0.96)
GNB	0.93	0.89	0.94	0.96
	(0.89, 0.97)	(0.85, 0.93)	(0.92, 0.96)	(0.94, 0.98)
Adaboost	0.95	0.96	**0.98**	0.97
	(0.93, 0.97)	(0.94, 0.98)	**(0.96, 1.00)**	(0.95, 0.99)

The best results are in bold.

**Figure 6:**
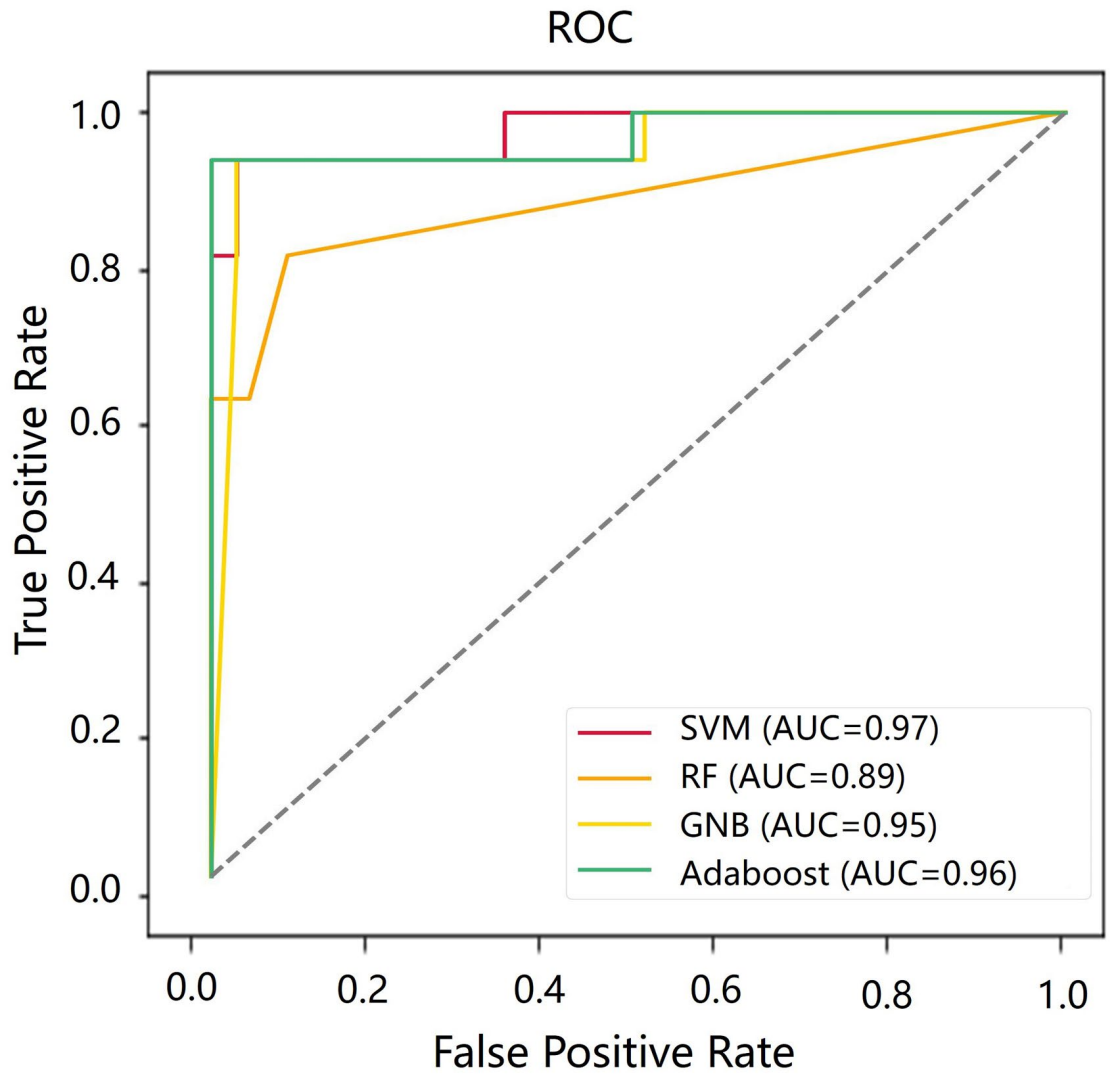
The receiver operation characteristics (ROC) curve of SVM in the test set.

Table 4 shows the classification results in the test set. For the performance of different classifiers, the SVM classifier got ACC = 0.95, SEN = 0.96, SPE = 0.94, and AUC = 0.97, which performs the best. In addition, we also use ROC and AUC values to evaluate different methods. In order to facilitate comparative analysis, we draw the ROC curves of different classification algorithms in the same coordinate graph, as shown in Figure 6. Among them, the ROC curve of SVM is closer to the upper left corner of the coordinate, which means the corresponding AUC value is the largest. Besides, the AUC value of GNB is significantly lower than the other three.

**Table 4:** Classification results of different classifiers in the test set.

Classifier	ACC	SEN	SPE	AUC
SVM	0.95	0.96	0.94	0.97
RF	0.84	0.86	0.88	0.89
GNB	0.91	0.92	0.91	0.95
Adaboost	0.92	0.93	0.94	0.96

## Discussion

In this study, we proposed a novel machine learning pipeline to automatically segment the hippocampus and classify AD from NC. The AL-SCF pipeline achieved excellent performance in hippocampus segmentation and AD classification. The total time of the whole pipeline was less than 1 min in the test set. Thus, our AL-SCF pipeline would be helpful to improve diagnostic accuracy and assist radiologists in clinical practice.

Our method first cropped the coronal T1 weighted images to eliminate the negative effects of the low percentage of hippocampal regions in the brain. We also adopted data augmentation in the segmentation task, which could improve the Dice of hippocampus segmentation. According to Table 2, data augmentation methods are significantly effective to improve the model performance when the image samples are limited. Besides, U2-net was transferred in the hippocampus segmentation from the target detection tasks. The model not only retains the characteristics of traditional U-net but also adds a deep supervision mechanism to learn low-dimensional and high-dimensional features. The convergence of the loss function can be accelerated by calculating the difference between the segmentation results and the Ground Truth of each layer. In previous studies, Sohail and Anwar (2022) and Liu et al. (2020) proposed simple applications for U-net, with Dice of 0.87 and 0.89, respectively. Our segmentation model achieved Dice of 0.93, better than the former results.

In this study, the features of original images and the wavelet-transformed higher-order features (Gillies et al., 2016; Yip and Aerts, 2016) were collected in the feature extraction part. Among them, the wavelet transform could concentrate the energy of the original image on a small portion of wavelet coefficients and provide more useful information for feature extraction (Feng and Ding, 2020). The hierarchical clustering algorithm was used for feature selection, which effectively avoid overfitting, and the subsequent LASSO algorithm further optimized the combined form and weights of the selected features. In addition, we used the grid search method (*λ* = 0.05, 0.10.0.0.60) and nested 5-fold cross-validation to determine the optimal LASSO hyperparameters *λ* and evaluate the performance of the model. Our classification results confirmed the effectiveness of the methods.

The majority of current studies (Liu et al., 2020; Nadal et al., 2020; Park et al., 2022) used deep learning networks in AD classification while we took the classical classifiers in machine learning, which is more interpretable and can find radiomics features associated with AD. Radiomic analysis has been applied to some AD studies. Zhang et al. (2012) indicated that 3D texture analysis could distinguish AD patients from the NC group, with classification ACC between 0.64 and 0.96 due to different ROI selections. Feng et al. (2018) built a support vector machine model which achieves an ACC = 0.87 (SPE = 0.89 and SEN = 0.84) in predicting AD vs. NC. The proposed classification models based on hippocampus radiomic features in our study showed high performance (ACC = 0.95 and AUC = 0.97) in distinguishing AD-NC.

In our study, we selected the hippocampus as the ROIs and the results showed that the radiomics features of the hippocampus can be used for the diagnosis of AD from normal controls. Furthermore, we found the shape features of the hippocampus (lateral maximum diameter and axial length) are highly correlated with AD. These are similar to the findings of Shen et al. (2012) and Nadal et al. (2020) used the shape descriptors from statistical shape models (SSM) as features to classify AD from normal control cases and discovered the shape of the hippocampus can provide valuable information for the diagnosis of AD. Nadal et al. presented a brain T1-weighted structural magnetic resonance imaging (MRI) biomarker which combined several individual MRI biomarkers such as volumetric measurements, hippocampal shape, and hippocampal texture. Their experiments showed that both common and uncommon individual MRI biomarkers contributed to the classification of AD.

However, our study still has some inevitable limitations. First, although the sample size of our study is relatively larger than that of some machine learning studies (Feng et al., 2021; Liu et al., 2022), the sample size is still relatively limited. Further studies are needed to conduct prospective multi-center studies in conjunction with multiple centers. Second, our study only investigated the effect of radiomics features in the hippocampus as a biomarker for AD, the application of other potential biomarkers for prediction could be analyzed in future studies.

## Conclusion

The AL-SCF pipeline is a non-invasive method that includes the automatic segmentation of the hippocampus and AD classification. The results suggest that micro-structural changes in the brain hippocampus region reflected by radiomic features can be a reliable method to assist radiologists in clinical practice. Furthermore, the proposed automatic segmentation and classification framework could be easily applied to other radiomics studies in the future.

## Data availability statement

The raw data supporting the conclusions of this article will be made available by the authors, without undue reservation.

## Ethics statement

The studies involving human participants were reviewed and approved by Huashan Hospital Institutional Review Board, Fudan University. Written informed consent for participation was not required for this study in accordance with the national legislation and the institutional requirements.

## Author contributions

KZ conducted the experiment, performed the data processing and analysis, and wrote and edited the manuscript. XLi, XLu, and HC collected the data, performed the data processing and analysis, and edited the manuscript. SP and RX collected the data and performed the data processing and analysis. DG supervised the whole study. All authors contributed to the article and approved the submitted version.

## Funding

This study was supported by the Greater Bay Area Institute of Precision Medicine (Guangzhou), Fudan University (KCH2310094, 21618), and Medical Engineering Joint Fund of Fudan University.

## Conflict of interest

The authors declare that the research was conducted in the absence of any commercial or financial relationships that could be construed as a potential conflict of interest.

## Publisher’s note

All claims expressed in this article are solely those of the authors and do not necessarily represent those of their affiliated organizations, or those of the publisher, the editors and the reviewers. Any product that may be evaluated in this article, or claim that may be made by its manufacturer, is not guaranteed or endorsed by the publisher.

## Footnotes

^1^ http://www.itksnap.org/

^2^ https://pytorch.org/

^3^ https://scikit-learn.org/
